# Targeted next generation sequencing in Italian patients with Usher syndrome: phenotype-genotype correlations

**DOI:** 10.1038/s41598-017-16014-z

**Published:** 2017-11-15

**Authors:** Chiara M. Eandi, Laura Dallorto, Roberta Spinetta, Maria Pia Micieli, Mario Vanzetti, Alessandro Mariottini, Ilaria Passerini, Francesca Torricelli, Camilla Alovisi, Cristiana Marchese

**Affiliations:** 1 Department of Surgical Sciences, Eye Clinic, Università degli Studi di Torino, Torino, 10122 Italy; 2 Low vision Unit, Ospedale Oftalmico Sperino Torino, Torino, 10122 Italy; 3 Inherited retinal dystrophies Unit, Azienda Ospedaliera Ordine Mauriziano Torino, Torino, 10128 Italy; 4 Diagnostic Genetic Unit, AOU Careggi Firenze, Firenze, 50134 Italy

## Abstract

We report results of DNA analysis with next generation sequencing (NGS) of 21 consecutive Italian patients from 17 unrelated families with clinical diagnosis of Usher syndrome (4 USH1 and 17 USH2) searching for mutations in 11 genes: *MYO7A, CDH23, PCDH15, USH1C, USH1G, USH2A*, *ADGVR1, DFNB31, CLRN1, PDZD7, HARS*. Likely causative mutations were found in all patients: 25 pathogenic variants, 18 previously reported and 7 novel, were identified in three genes (*USH2A*, *MYO7A*, *ADGRV1*). All USH1 presented biallelic MYO7A mutations, one USH2 exhibited *ADGRV1* mutations, whereas 16 USH2 displayed *USH2A* mutations. USH1 patients experienced hearing problems very early in life, followed by visual impairment at 1, 4 and 6 years. Visual symptoms were noticed at age 20 in a patient with homozygous novel *MYO7A* missense mutation c.849G > A. USH2 patients’ auditory symptoms, instead, arose between 11 months and 14 years, while visual impairment occurred later on. A homozygous c.5933_5940del;5950_5960dup *in USH2A* was detected in one patient with early deafness. One patient with homozygous deletion from exon 23 to 32 in *USH2A* suffered early visual symptoms. Therefore, the type of mutation *in USH2A* a*nd MY*O7A genes seems to affect the age at which both auditory and visual impairment occur in patients with USH.

## Introduction

Usher Syndrome (USH) is a syndromic inherited retinal dystrophy (IRD). It is a clinically and genetically heterogeneous autosomal recessive disorder, characterized by retinitis pigmentosa (RP)^1–3^ and bilateral sensorineural deafness, with or without vestibular dysfunction.

USH has a prevalence of 3.2 to 6.2 cases per 100000, thus representing the leading genetic cause of combined hearing and vision loss^4,5^. Three different clinical subtypes have been so far identified based on hearing, vestibular and visual symptoms^6^. Type 1 (USH1) is the most severe form of USH, characterized by severe congenital deafness, vestibular dysfunction and prepubertal RP onset^7^. Deafness is less severe in USH type 2 (USH2), for which the vestibular function remains normal, while RP symptoms generally occur during puberty^7^. Finally, USH type 3 (USH3) is characterized by post lingual hearing loss, while the age of RP occurrence is offset between the second to fourth decade and can be subject to variable vestibular dysfunctions^6^. Sixteen loci and thirteen disease-causing genes associated with USH have been so far identified (https://sph.uth.edu/retnet/), thus demonstrating the wide genetic heterogeneity of this syndrome. Such heterogeneity makes molecular diagnosis with Sanger sequencing of single genes rather challenging, since this particular diagnostic strategy, although accurate, requires a lot of time and resources^6^. Microarray technology for the simultaneous detection of known mutations in Usher related genes, instead, has proven to perform poorly, proving diagnostic efficiency as low as 12%, when applied on the Italian population^8^. Nevertheless, thanks to the recent availability of next generation sequencing (NGS) in routine genetic testing, it is now possible to simultaneously screen an increasing number of selected genes (targeted NGS), as well as the whole exome or the entire genome. So far, NGS has proven to be a rapid and cost-effective diagnostic method, which is also a useful tool for the identification of novel disease-causing genes^9^. Targeted NGS is being currently applied on patients affected by hereditary diseases with high gene heterogeneity, such as Usher syndrome and, both syndromic and non-syndromic, inherited retinal dystrophies^10–13^.

Deeper understanding of the genetic basis allows further analysis of possible genotype-phenotype correlations. Besides being useful for genetic counselling, such knowledge base is fundamental to an accurate clinical diagnosis and prognosis of individual patients and thus represents an invaluable tool for the development of personalized treatments.

The report describes the phenotypes associated with the mutations detected during genetic testing with targeted NGS. The tests were carried out on a group of 21 consecutive Italian patients from 17 unrelated families affected by Usher syndrome who were seeking genetic counselling.

## Methods

### Patients

**Figure 1:**
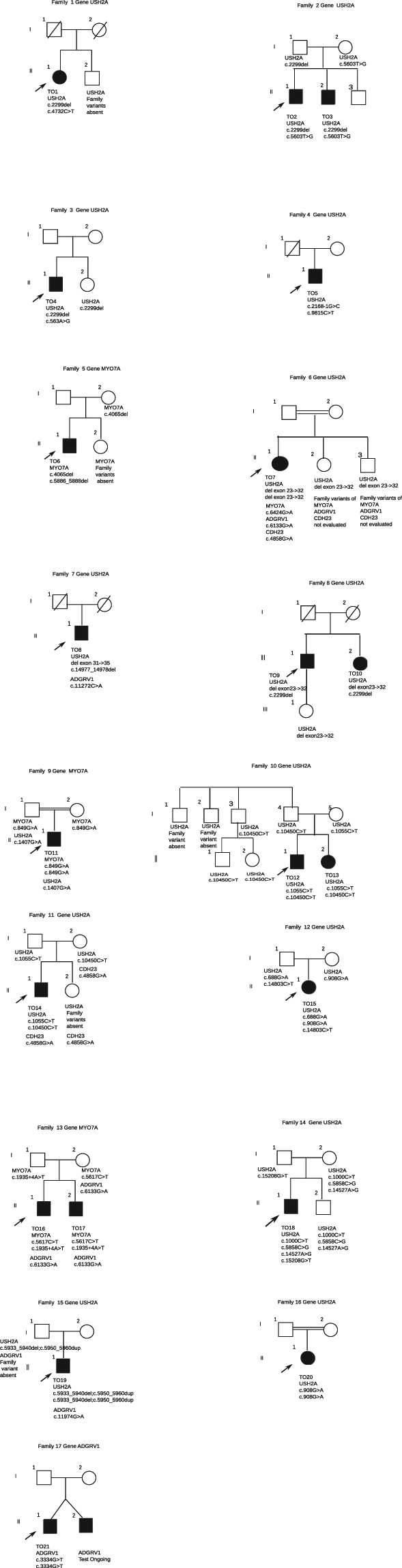
Pedigrees of the families included in the study.

This is a retrospective study. The study comprises 21 consecutive Italian patients (15 males and 6 females) from 17 unrelated families with a clinical diagnosis of Usher syndrome who were seeking genetic counselling at the Mauriziano Hospital in Torino, Italy. The pedigrees of the 17 families are reported in Fig. 1.

The data collection complies with the Italian law. The study was conducted in accordance with the provisions stated in the Declaration of Helsinki (59th World Medical Association General Assembly; Seoul, Korea; October 2008).

All the patients and their relatives were duly informed about the advantages and limitations of the test and were required to sign informed consent. Detailed medical, personal and family history was obtained from the patients and their relatives, specifically recording the age of onset of deafness, visual deficiency and equilibrium impairment.

In most cases, the available ophthalmological clinical data were composed by slit-lamp anterior segment and fundus examinations, best corrected visual acuity, Goldmann applanation tonometry, electroretinogram (ERG), visual evoked potentials (VEP), visual field test, optic coherence tomography (OCT). A few patients had also sustained colour vision tests, fundus autofluorescence and fluorescein angiography.

### DNA analysis

Within the framework of the Italian rare diseases registry, the diagnostic genetic unit of AOU Careggi in Firenze offers National Health System patients free genetic testing for IRD. Genetic testing is mainly requested to confirm clinical diagnosis, for genetic counselling for patients and their families and to allow participation in gene therapy trials.

Genomic DNA was isolated from peripheral leukocytes, using the QiaSymphony DNA Blood Midi kit on the QIAsymphony SP workstation (Qiagen), according to the manufacturer’s protocol.

A custom Haloplex panel was designed using Agilent’s online SureDesign tool (https://earray.chem.agilent.com/suredesign/index.htm), Targeted NGS of coding regions and exon-intron junctions of a panel of 11 genes were performed: *MYO7A* (MIM 276903)*, CDH23* (MIM 605516)*, PCDH15* (MIM 605514)*, USH1C* (MIM 605242)*, USH1G* (MIM 607696)*, USH2A* (MIM 608400), *ADGVR1* (MIM 602851)*, DFNB31* (MIM 607928)*, CLRN1* (MIM 606397)*, PDZD7* (MIM 612971)*, HARS* (MIM 142810). Genes *ABDH12* (MIM 613599,612674), *CEP250* (MIM 609689) and *CIB2* (MIM 614869) were not included in the panel because mutations in these genes have only been recorded in a few non-Caucasian families. *PDZD7* gene was included because it has been suggested to be a modifier gene in subjects with *USH2A* mutations and potentially involved with *ADGRV1* in digenic USH2^14^.

The target regions were captured using the Agilent HaloPlex Target Enrichment System Kits for Illumina Sequencing following Agilent protocols. The captured target libraries were amplified by PCR, quality controlled and quantified using the BioAnalyzer 2100 (Agilent Technologies, Inc. Santa Clare, CA). Equimolar amounts of differentially indexed samples were pooled before pair-ended sequencing at 300 cycles on the Illumina MiSeq platform (Illumina Inc., San Diego, CA, USA). In addition, the deep intronic variant (c.7595-2144A > G) in intron 40 of *USH2A* gene was searched^15^.

The criteria used to distinguish new mutations from polymorphisms is ExAC frequency. We filtered variants with a MAF < 0.05. All the new mutations reported in this study are not validated at RNA and protein levels.

The new mutations reported were investigated in 48 healthy subjects (20 females, 28 males) with Sanger sequencing.

For the new point mutations leading to aminoacid substitution, pathogenicity predictions from Bioinformatic tools SIFT, PhyloP, AGVGD, MutationTaster and Polyphen2 were compared.

For intronic mutations, although our laboratory cannot validate that the mutations observed indeed affect the splicing process, the bioinformatics tools available predicted all of them to be pathogenic; furthermore the variants were at the exon-intron junction and such variants both at RNA level and in classical genetics are reported to affect the splicing process.

The presence of all pathogenic and likely pathogenic variants detected was confirmed with Sanger sequencing, processed with the automated Core System (Beckman Coulter, Fullerton, CA). After purification, amplicons were sequenced on the 3730 DNA Analyzer (ABI, Foster City, CA). The sequences were assembled and analyzed using SeqScape software (ABI). Variants of unknown pathogenicity were interpreted with Alamut 2.6 (Interactive Biosoftware, Rouen, France), a decision-support software application for medical molecular genetics. The software relies on web-based prediction software, such as Align-GVGD, SIFT, PolyPhen, Mutation Taster (hosted by Interactive Biosoftware). Note that Alamut 2.6 scoring systems provide a predictive evaluation only for missense variants. In selected patients multiplex ligation-dependent probe amplification (MLPA) was also performed. The MLPA reaction (P361-A1/P362-A2 SALSA MLPA kit; MRC Holland, Amsterdam, The Netherlands) was performed according to the manufacturer’s recommendations. One microliter of each reaction product was separated on a POP-7 polymer with capillary electrophoresis using the 3730 DNA Analyzer (ABI). Freely available software provided by MRC Holland was used to analyze the MLPA data (Coffalyser; MRC Holland).

When relatives were available (14 families), segregation analysis was performed.

## Results

### DNA analysis

DNA analysis results were in accordance with the diagnosis for all patients clinically diagnosed with USH. The average of sequencing depth was about >99.9%. We obtained about 70 variants for each sample. We filtered for function and frequency according to ACMG guidelines^16^. We obtained about 1–3 variants for sample that were validated by Sanger sequencing.

Likely causative mutations were identified in three Usher related genes: *USH2A*, *MYO7A, ADGRV1*. For the causative mutations found in this study, reported and novel, there aren’t neither *in vivo* functional experiments showing that the mutations cause the USH phenotype, nor *in vitro* experiments showing that the mutations will cause genetic dysfunction.

The results are reported in Table 1. Further heterozygous mutations in Usher related genes were observed in nine patients (Table 2). In Table 3 the frequency in our normal population of the new mutation and the pathogenecity prediction from Bioinformatic tools SIFT, PhyloP, AGVGD, MutationTaster and Poluphen2 for the new point mutations leading to aminoacid substitution, are reported. In Fig. 1 the pedigree of the 17 families and the genotypes of the patients and of their relatives are reported.

**Table 1:** Patients’ phenotypes and mutations identified in Usher genes.

Family	Patient	Age*	Sex	Onset HL (yrs)	Onset VI (yrs)	Gene	Mutation	Protein		Reported	Segregation
1	TO1	47	F	14	20	USH2A	c.2299del	(p.Glu767Serfs*21)	Heterozygous	Reported	YES
						USH2A	c.4732C > T	(p.Arg1578Cys)	Heterozygous	Reported	
2	TO2	35	M	7	16	USH2A	c.2299del	(p.Glu767Serfs*21)	Heterozygous	Reported	YES
						USH2A	c.5603T > G	(p.Phe1868Cys)	Heterozygous	Reported	
2	TO3	29	M	6	16	USH2A	c.2299del	(p.Glu767Serfs*21)	Heterozygous	Reported	YES
						USH2A	c.5603T > G	(p.Phe1868Cys)	Heterozygous	Reported	
3	TO4	17	M	3	10	USH2A	c.2299del	(p.Glu767Serfs*21)	Heterozygous	Reported	YES
						USH2A	**c.563A > G**	**(p.Tyr188Cys)**	Heterozygous	**Novel**	
4	TO5	58	M	7	12	USH2A	c.2168-1G > C	p?	Heterozygous	Reported	Not done
						USH2A	c.9815C > T	(p.Pro3272Leu)	Heterozygous	Reported	
5	TO6	32	M	Cong	6	MYO7A	c.4065del	(p.His1355Glnfs*44)	Heterozygous	Reported	YES
						MYO7A	c.5886_5888del	(p.1963del)	Heterozygous	Reported	
6	TO7	21	F	2	10	USH2A	Del exon 23 > 32	p?	Homozygous	Reported	YES
7	TO8	54	M	6	30	USH2A	**Del exon 31 > 35**	**p?**	Heterozygous	**Novel**	Not done
						USH2A	c.14977_14978del	(p. Phe4993Profs*7)	Heterozygous	Reported	
8	TO9	48	M	6	8	USH2A	Del exon23 > 32	p?	Heterozygous	Reported	YES
						USH2A	c.2299del	(p.Glu767Serfs*21)	Heterozygous	Reported	
8	TO10	46	F	7	18	USH2A	Del exon 23 > 32	p?	Heterozygous	Reported	YES
						USH2A	c.2299del	(p.Glu767Serfs*21)	Heterozygous	Reported	
9	TO11	31	M	3 mo	19	MYO7A	**c.849 G > A**	**(p.Met283Ile)**	Homozygous	**Novel**	YES
10	TO12	16	M	3	16	USH2A	c.1055C > T	(p.Thr352Ile)	Heterozygous	Reported	YES
						USH2A	c.10450C > T	(p.Arg3484*)	Heterozygous	Reported	
10	TO13	20	F	3	18	USH2A	c.1055C > T	(p.Thr352Ile)	Heterozygous	Reported	YES
						USH2A	c.10450C > T	(p.Arg3484*)	Heterozygous	Reported	
11	TO14	45	M	7	20	USH2A	c.269A > G	(p.Tyr90Cys)	Heterozygous	Reported	YES
						USH2A	c.14977_14987del	(p.Phe4993Profs*7)	Heterozygous	Reported	
12	TO15	33	F	7	7	USH2A	c.908G > A	(p.Arg303His)	Heterozygous	Reported	YES
						USH2A	c.14803C > T	(p.Arg4935*)	Heterozygous	Reported	
13	TO16	12	M	Cong	1	MYO7A	c.5617C > T	(p.Arg1873Trp)	Heterozygous	Reported	YES
						MYO7A	**c.1935 + 4A > T**	**p?**	Heterozygous	**Novel**	
13	TO17	23	M	18mo	4	MYO7A	c.5617C > T	(p.Arg1873Trp)	Heterozygous	Reported	YES
						MYO7A	**c.1935 + 4A > T**	**p?**	Heterozygous	**Novel**	
14	TO18	30	M	6	16	USH2A	c.1000C > T	(p.Arg334Trp)	Heterozygous	Reported	YES
						USH2A	**c.15208G > T**	(p.Glu5070*)	Heterozygous	**Novel**	
15	TO19	20	M	11mo	17	USH2A	c.5933_5940del	p.Pro1978Glnfs*5	Homozygous	Reported	YES
						USH2A	**c.5950_5960dup**	p.Tyr1987*	Homozygous	**Novel**	YES
16	TO20	28	F	4	15	USH2A	c.908G > A	(p.Arg303His)	Homozygous	Reported	YES
17	TO21	53	M	5	38	ADGRV1	**c.3334G > T**	p.Glu1112*	Homozygous	**Novel**	Not done

HL = hearing loss; VI = visual impairment; ^*^Age at counselling and genetic testing; M = male; F = female; yrs = years; mo = months; cong = congenital.

**Table 2:** Additional variants in Usher genes.

Family	Patient	Age*	Sex	Onset HL (yrs)	Onset VI (yrs)	Gene	Mutation	Protein		Reported	Segregation
6	TO7	21	F	2	10	MYO7A	c.6424G > A	p.Asp2142Asn	Heterozygous	Reported	YES
						ADGRV1	c.6133G > A	p.Gly2045Arg	Heterozygous	Novel	
						CDH23	c.4858G > A	p.Val1620Met	Heterozygous	Reported	
7	TO8	54	M	6	30	ADGRV1	c.11272C > A	(p.Gln3758Lys)	Heterozygous	Novel	Not done
9	TO11	31	M	3 mo	19	USH2A	c.1407G > A	(p.Gly4692Arg)	Heterozygous	Reported	YES
11	TO14	45	M	7	20	CDH23	c.4858G > A	(p.Val1620Met)	Heterozygous	Reported	YES
12	TO15	33	F	7	7	USH2A	c.688G > A	(p.Val230Met)	Heterozygous	Reported	YES
13	TO16	12	M	Cong	1	ADGRV1	c.6133G > A	(p.Gly2045Arg)	Heterozygous	Novel	YES
13	TO17	23	M	18mo	4	ADGRV1	c.6133G > A	(p.Gly2045Arg)	Heterozygous	Novel	YES
14	TO18	30	M	6	16	USH2A	c.5858C > G	(p.Ala1953Gly)	Heterozygous	Reported	YES
						USH2A	c.14527 A > G	(p.Arg4843Gly)	Heterozygous	Novel	
15	TO19	20	M	11mo	17	ADGRV1	c.11974G > A	(p.Asp3992 Asn)	Heterozygous	Novel	YES

HL = hearing loss; VI = visual impairment; ^*^Age at counselling and genetic testing; M = male; F = female; yrs = years; mo = months; cong = congenital

**Table 3:** New mutations: frequency in control population and pathogenicity prediction

Change in		dbSNP		Prediction algorithms				
Nucleotide	Protein	ID	MAF (%)	phyloP	SIFT	PolyPhen2	Mutation Taster	Align GVGD
***USH2A***								
c.563A > G	p.Tyr188Cy	NA	0	Moderately conserved nucleotide	Deleterious	Probably damaging	Disease causing	C0
del exon 31- > 35	p.?			—	—	—	—	—
c.15208G > T	p.Glu5070*	NA	0	—	—	—	—	—
c.5950_5960dup	p.Tyr1987*	NA	0	—	—	—	—	—
***MYO7A***				—	—	—	—	—
c.849 G > A	p.Met283Ile	NA	0	Moderately conserved nucleotide	Tolerated	Benign	Disease causing	C0
c.1935 + 4 A > T	p.?	NA	0	—	—	—	—	—
***ADGRV1***								
c.3334 G > T	p.Glu1112*	NA	0	—	—	—	—	—

MAF = minor allele frequency; SIFT = sorting intolerant from tolerant; PolyPhen2 = polymorphism phenotyping; NA = not applicable.

### Phenotype

Details of the phenotypes are reported in Table 4.

**Table 4:** Clinical characteristics.

Patient	Age*	Sex	BCVA	Anatomic changes	GVF	CME	Hearing changes	Vestibular function	Other
TO1	47	F	RE: 0.05 LE: 0.04	Bilateral cataract, RP sine pigmento,	Residual bilateral 10°	Absent	Bilateral progressive pantonal neurosensory hearing loss	Subjective equilibrium impairment in the 5^th^ decade	
TO2	35	M	RE: 0.2 LE: 0.25	Bilateral cataract	N/A	Macular hole in RE	Bilateral progressive neurosensory hearing loss	No subjective equilibrium impairment	
TO3	29	M	RE: 0.2 LE: 0.25	Bilateral cataract, bilateral pigment deposits	Residual bilateral 10°	N/A	Bilateral non progressive post lingual hearing loss	No subjective equilibrium impairment	
TO4	17	M	RE: 0.4 LE: 0.6	Bilateral pigment deposits	Residual bilateral 10°	N/A	Bilateral neurosensory hearing loss	No subjective equilibrium impairment Vestibular test normal	Two episodes of absence seizure
TO5	58	M	RE: 0.8 LE: 0.6	Bilateral cataract, bilateral pigment deposits	Residual bilateral 20°	N/A	Bilateral neurosensory median and high frequencies hearing loss	No subjective equilibrium impairment	
TO6	32	M	RE: 0.3 LE: 0.3	Bilateral cataract, bilateral pigment deposits	RE: Residual isle in temporal-superior, LE: reduction of sensibility and nasal deficit	N/A	Bilateral congenital hearing loss	Delayed walking.Vestibular test abnormal	
TO7	21	F	RE: 0.4 LE: 0.2	Bilateral pigment deposits, vitreo-macular traction	Residual bilateral < 10°	Present bilateral	Bilateral congenital moderate/profound hearing loss	Delayed walking Vestibular test abnormal	
TO8	54	M	RE: 0.8 LE: 0.4	Bilateral cataract, bilateral pigment deposits, peripapillary atrophy, macular pucker,	RE: Residual central 6–10° LE: Residual central 10–15°	N/A	Bilateral pan-tonal non progressive severe neurosensory hearing loss	Vestibular test abnormal	Migraine episodes
TO9	48	M	RE: 0.5 LE: 0.7	Bilateral cataract, bilateral pigment deposits, peripapillary atrophy.	Residual bilateral 10°	N/A	Bilateral pan tonal severe neurosensory hearing loss	Vestibular test abnormal	
TO10	46	F	N/A	N/A	N/A	N/A	Bilateral moderate hearing loss	Subjective equilibrium impairment	
TO11	31	M	RE: 0.95 LE: 0.95	Bilateral pigment deposits	Residual bilateral 20°	Present	Bilateral congenital progressive severe hearing loss, bilateral cochlear implant	No subjective equilibrium impairment. Walked at 13 months	
TO12	16	M	RE: 1 LE: 1	Bilateral pigment deposits and atrophic areas	N/A	Present bilateral	Bilateral congenital hearing loss, hearing aids	No subjective equilibrium impairment	
TO13	20	F	RE: 0.9 LE: 0.5	Bilateral pigment deposits, optic nerve head drusen	Residual bilateral 20°	Present	Bilateral mild hearing loss	No subjective equilibrium impairment	*GJB2* wild-type Headache, recurrent sinusitis and bronchitis
TO14	45	M	RE: 1 LE: 1	Bilateral cataract, bilateral pigment deposits	Residual bilateral 10°	Absent	Bilateral mild progressive neurosensory hearing loss	Subjective equilibrium impairment	Neonatal jaundice
TO15	33	F	N/A	Bilateral pigment deposits	Residual bilateral 20°	Absent	Bilateral mild high frequencies neurosensory hearing loss	No subjective equilibrium impairment. Vestibular test normal	Migraine. *CPT2* gene mutations associated myopathy
TO16	12	M	RE: 0.7 LE: 0.5	Abnormal macular reflex, pale optic disk, no pigment deposit	N/A	Present LE	Bilateral congenital hearing loss, cochlear implant	Delayed walking (18 months)	Pervasive development disorder
TO17	23	M	RE: 1 LE: 0.15	Bilateral cataract, bilateral pigment deposits, peripheral retinal teleangectasia, vitreous hemorrhage in LE, optic disk drusen	Residual bilateral 10°	Present LE	Bilateral congenital severe hearing loss, cochlear implant	Delayed walking (24 months) Vestibular test abnormal	Scleral buckling and vitrectomy in LE for retinal detachment. *GJB2* wild-type
TO18	30	M	RE: 0.55 LE: 0.45	Bilateral pigment deposits	Reduced sensitivity with paracentral scotomatous areas	Present bilateral	Bilateral progressive neurosensory hearing loss,hearing aids	No subjective equilibrium impairment	Liver angioma *GJB2* wild-type
TO19	20	M	RE: 0.9 LE: 0.8	Bilateral pigment deposits	Residual bilateral 10°	N/A	Bilateral progressive mild post lingual neurosensory hearing loss	No subjective equilibrium impairment	Dorsal scoliosis
TO20	28	F	RE: 0.5 LE: 0.5	Bilateral cataract, bilateral pigment deposits	Residual bilateral 10°	Absent	Bilateral progressive mild post lingual neurosensory hearing loss	Normal	Several episodes of loss of consciousness, abnormal EEG
TO21	53	M	RE: 0.5 LE: 0.6	Bilateral cataract. bilateral pigment deposits	Residual bilateral 10°	N/A	Bilateral progressive severe post lingual neurosensory hearing loss	No subjective equilibrium impairment	

RE, right eye; LE, left eye; BCVA, best corrected visual acuity; GVF, Goldmann Visual Field; CME, cystoid macular edema; N/A, not available; EEG; electroencephalogram.

## Discussion

This report retrospectively analyses the clinical and genetic data of 21 consecutive Italian patients from 17 unrelated families affected by Usher syndrome undergoing genetic analysis by targeted NGS of 11 genes (*MYO7A, CDH23, PCDH15, USH1C, USH1G*, *USH2A*, *ADGVR1, DFNB31, CLRN1, PDZD7, HARS*). USH2 syndrome was the most frequent clinical diagnosis, accounting for 81% of the patients, while USH1 was diagnosed in 19%. None of the patients were affected by USH3.

24 likely pathogenic variants - 17 previously reported and 7 novel - were identified in three genes (*USH2A*, *MYO7A*, *ADGRV1*). Five mutations were detected in *MYO7A*, two of which were novel; 18 mutations were identified in *USH2A* (4 of which novel) and one novel homozygous mutation was identified in *ADGRV1*. Additional variants with uncertain pathogenic significance in *USH2A*, *MYO7A*, *ADGRV1*, *CDH23* genes were further identified in 9 patients. In these patients, the additional variants were not considered to be the main causative mutations because two other causative variants were present (reported or novel but with a strong impact at the protein level). We cannot exclude a modifier role for these uncertain variants.

Genetic testing result was in accordance with previous clinical diagnosis or clinical suspicion for all patients. All subjects with USH1 or suspected USH1 displayed biallelic *MYO7A* mutations and all subjects with USH2 presented at least two *USH2A* or two *ADGRV1* mutations. Such results are in line with previous studies that report *MYO7A* and *USH2A* gene mutations being among the most frequent causes of USH1 and USH2^17^. The frameshift mutation c.2299delG (p.Glu767Serfs*21) in the *USH2A* gene was the most frequent in our cohort, since it was detected in 6 patients from four unrelated families. This mutation is quite frequent (0.16 to 0.44) in several cohorts of patients^18–21^. According to current literature, this is the most common mutation in European patients, accounting for approximately 30% of all European cases of *USH2A* ^22^. The high frequency of such mutation has been reported in several different populations and was proven to be the result of an ancestral mutation that has then spread throughout Europe and other continents due to migratory movements^23^.

Segregation analysis revealed three patients (TO15, TO18 and TO19) with a *USH2A* allele carrying more than one mutation. This finding underlines the importance of performing segregation analysis on patients suffering from recessive disorders to identify the exact genotype. Although such thoroughness adds up to the costs, it is essential for genetic counselling and reliable family risk evaluation.

All subjects with USH1 and biallelic *MYO7A* mutations were diagnosed with deafness and vestibular function impairment within their first 18 months of life. This lead to an initial clinical diagnosis of myopathy with neurodevelopmental delay in one patient (TO17), while the brother (TO16) was diagnosed with a pervasive developmental disorder. For all patients, deafness was initially attributed to a possible prenatal or postnatal infection. Three patients were tested in early childhood for *GJB2* gene mutations, since this gene is deemed to be the leading cause for hereditary deafness within the European population^24^. At the time of diagnosis, the absence of pathogenic variants in *GJB2* gene had, in fact, misled the physician in reinforcing the hypothesis of an infective aetiology of deafness. The parents of two of these patients, having been reassured about a low risk of recurrence, had a second affected child. This further stresses the fundamental importance of providing an exact diagnosis to children affected by deafness. Such diagnosis can be accomplished by offering targeted NGS for syndromic and non-syndromic deafness-related genes whenever prenatal or postnatal infective aetiology is not documented.

Visual symptoms have also proven to occur at an early age, ranging from 1 to 6 years, hence well before the age of 10, as generally observed in USH1 subjects^25^. Only patient TO11 had a late onset of visual symptoms, which went unnoticed until he was 19. This patient has a novel homozygous missense mutation in *MYO7A* gene that might have a milder effect on retinal function.

A recent study on the Italian population confirms that hearing and visual impairment generally occur at an earlier age in patients carrying MYO7A mutations compared to those carrying USH2A mutations. The mean age for hearing loss and visual symptoms is generally between 5 ± 1 months and 16 ± 3 years respectively^26^.

Patients with USH2 carrying *USH2A* or *ADGRV1* mutations were diagnosed deaf and displayed visual symptoms at an older age compared to subjects with *MYO7A* mutations. Deafness usually occurred between 11 months and 14 years (mean age 5 years) within the reported range 8 months – 31 years^27^, whereas visual impairment generally onsets later, among 10 to 38 year old, (mean 15 ± 8.4 years) again within the reported age range 8–76 (average age 35.5)^27^. Two homozygous c.5933_5940del and 5950_5960dup mutations in *USH2A* were detected in patient TO19, whose deafness onset was recorded when he was 11 months old and whose visual symptoms were noticed only later when he was 17 years old.

The age at which the patients with USH2 in our cohort lamented symptoms was generally earlier than what a recent study on Italian patients with USH^17^ reports. The study comprises 36 patients (three USH1 and 33 USH2) and reports the average age of visual symptoms onset to be 17.5 ± 8.8 years. OCT revealed macular oedema in 29% of the patients in our study, a comparable percentage of cystoid macular lesions (from 28 to 49% in different studies) were similarly reported in studies on patients with RP^28–30^.

In conclusion, patients with USH exhibited clinical severity, which appears to be related to the mutated gene and to the specific type of mutation. Homozygosity for deletion from exon 23 to 32 and homozygosity for c.5933_5940del and c.5950_5960dup in *USH2A* were associated with a severe phenotype. It is known that mutations in *USH2A* can lead either to USH2 or to non-syndromic RP. Mutations carried by USH2 patients, tend to be more severe than those found in non-syndromic RP patients^31,32^. Therefore, there is evidence that, even within the USH2 phenotype, there ought be a severity gradient depending on the specific mutation^33,34^. We also observed a *MYO7A* biallelic mutation that was generating a phenotype with vestibular dysfunction, though it entailing milder hearing and visual symptoms. The role of additional heterozygous mutations in other related Usher genes remains to be further investigated.

These results, thus, provide useful data not only for tailored genetic counselling but they also provide additional clues for early clinical diagnosis of patients with Usher syndrome.

Nevertheless, this study present some limits. The heterogeneity of the clinical information available, in fact, did not allow for statistical analysis.

## References

[1] Keats BJ, Corey DP (1999). The Usher syndromes. Am J Med Genet..

[2] Saihan Z, Webster AR, Luxon L, Bitner-Glindzicz M (2009). Update on Usher syndrome. Curr Opin Neurol..

[3] Bonnet C, El-Amraoui A (2012). Usher syndrome (sensorineural deafness and retinitis pigmentosa): pathogenesis, molecular diagnosis and therapeutic approaches. Curr Opin Neurol..

[4] Kimberling WJ (2010). Frequency of Usher syndrome in two paediatric populations: implications for genetic screening of deaf and hard of hearing children. Genet. Med..

[5] Lenarduzzi S (2015). Usher syndrome: an effective sequencing approach to establish a genetic and clinical diagnosis. Hear Res..

[6] Mathur P, Yang J (2015). Usher syndrome: Hearing loss, retinal degeneration and associated abnormalities. Biochim Biophys Acta..

[7] Lentz, J., Keats, B. J. Usher syndrome type II, in *GeneReviews®* (ed Pagon, R. A) (Seattle,1993–2017).

[8] Vozzi D (2011). Molecular epidemiology of Usher syndrome in Italy. Mol Vis..

[9] Bras J (2012). Use of next-generation sequencing and other whole-genome strategies to dissect neurological disease. Nat Rev Neurosci..

[10] Huang XF (2013). Targeted exome sequencing identified novel *USH2A* mutations in Usher syndrome families. PLoS One..

[11] Wang G, Liu Y, Zhu D, Klau GW, Feng W (2015). Bioinformatics Methods and Biological Interpretation for Next-Generation Sequencing Data. Biomed Res Int..

[12] van El CG (2013). Whole-genome sequencing in health care: recommendations of the European Society of Human Genetics. Eur J Hum Genet..

[13] Bonnet C (2016). An innovative strategy for the molecular diagnosis of Usher syndrome identifies causal biallelic mutations in 93% of European patients. Eur J Hum Genet..

[14] Ebermann I (2010). PDZD7 is a modifier of retinal disease and a contributor to digenic Usher syndrome. J Clin Invest..

[15] Vaché C (2012). Usher syndrome type 2 caused by activation of an USH2A pseudoexon: implications for diagnosis and therapy. Hum Mutat..

[16] Richards S (2015). Standards and Guidelines for the Interpretation of Sequence Variants: A Joint Consensus Recommendation of the American College of Medical Genetics and Genomics and the Association for Molecular Pathology. Genet Med..

[17] Sodi A (2014). *MYO7A* and *USH2A* gene sequence variants in Italian patients with Usher syndrome. Mol Vis..

[18] Beneyto MM (2000). Prevalence of 2314delG mutation in Spanish patients with Usher syndrome type II (USH2). Ophthalmic Genet..

[19] Dreyer B (2000). Identification of novel *USH2A* mutations: implications for the structure of *USH2A* protein. Eur J Hum Genet.

[20] Liu XZ (1999). A mutation (2314delG) in the Usher syndrome type IIA gene: high prevalence and phenotypic variation. Am J Hum Genet..

[21] Weston MD (2000). Genomic structure and identification of novel mutations in Usherin, the gene responsible for Usher syndrome type IIa. Am J Hum Genet..

[22] Aller E (2004). Genetic analysis of 2299delG and C759F mutations (*USH2A*) in patients with visual and/or auditory impairments. Eur J Hum Genet..

[23] Dreyer B (2001). A common ancestral origin of the frequent and widespread 2299delG *USH2A* mutation. Am J Hum Genet..

[24] Smith, R.J.H., Jones, M.K.N. Nonsyndromic Hearing Loss and Deafness, DFNB1. In *GeneReviews®* (Pagon, R.A. *et al*.) (Seattle,1993–2017).

[25] Gorlin RJ, Tilsner TJ, Feinstein S, Duvall AJ (1979). Usher’s syndrome type III. Arch Otolaryng..

[26] Testa, F. *et al*. Clinical presentation and disease course of usher syndrome because of mutations in MYO7A or USH2A. *Retina*. (2016). [Epub ahead of print]. 10.1097/IAE.0000000000001389 27828912

[27] Abadie C (2012). Audiological findings in 100 USH2 patients. Clin Genet..

[28] Ozdemir H, Karacorlu M, Karacorlu S (2005). Intravitreal triamcinolone acetonide for treatment of cystoid macular oedema in patients with retinitis pigmentosa. Acta Ophthalmol Scand..

[29] Hajali M, Fishman GA, Anderson RJ (2008). The prevalence of cystoids macular oedema in retinitis pigmentosa patients determined by optical coherence tomography. Br J Ophthalmol..

[30] Lai YH, Capasso JE, Kaiser R, Levin AV (2016). Intraretinal cystoid spaces in a patient with retinitis pigmentosa due to mutation in the MAK gene. Ophthalmic Genet..

[31] Wang F (2014). Next generation sequencing-based molecular diagnosis of retinitis pigmentosa: identification of a novel genotype-phenotype correlation and clinical refinements. Hum Genet..

[32] Jiang L (2015). Comprehensive molecular diagnosis of 67 Chinese Usher syndrome probands: high rate of ethnicity specific mutations in Chinese USH patients. Orphanet J Rare Dis..

[33] Yang J (2010). Ablation of whirlin long isoform disrupts the USH2 protein complex and causes vision and hearing loss. PLoS Genet..

[34] Pan L, Zhang M (2012). Structures of usher syndrome 1 proteins and their complexes. Physiology (Bethesda)..

